# *In Vitro* Neural Differentiation of Bone Marrow Mesenchymal Stem Cells Carrying the FTH1 Reporter Gene and Detection with MRI

**DOI:** 10.1155/2018/1978602

**Published:** 2018-06-26

**Authors:** Tong Mu, Yong Qin, Bo Liu, Xiaoya He, Yifan Liao, Jun Sun, Jiawen Qiu, Xiaomeng Li, Yi Zhong, Jinhua Cai

**Affiliations:** ^1^Department of Radiology, Children's Hospital of Chongqing Medical University, Chongqing 400014, China; ^2^Ministry of Education Key Laboratory of Child Development and Disorders, Chongqing 400014, China; ^3^Key Laboratory of Pediatrics in Chongqing, Chongqing 400014, China; ^4^Chongqing International Science and Technology Cooperation Center for Child Development and Disorders, Chongqing 400014, China; ^5^Department of Radiology, Mianyang Central Hospital, Mianyang 621000, China

## Abstract

Magnetic resonance imaging (MRI) based on the ferritin heavy chain 1 (FTH1) reporter gene has been used to trace stem cells. However, whether FTH1 expression is affected by stem cell differentiation or whether cell differentiation is affected by reporter gene expression remains unclear. Here, we explore the relationship between FTH1 expression and neural differentiation in the differentiation of mesenchymal stem cells (MSCs) carrying FTH1 into neuron-like cells and investigate the feasibility of using FTH1 as an MRI reporter gene to detect neurally differentiated cells. By inducing cell differentiation with all-trans retinoic acid and a modified neuronal medium, MSCs and MSCs-FTH1 were successfully differentiated into neuron-like cells (Neurons and Neurons-FTH1), and the neural differentiation rates were (91.56±7.89)% and (92.23±7.64)%, respectively. Neuron-specific markers, including nestin, neuron-specific enolase, and microtubule-associated protein-2, were significantly expressed in Neurons-FTH1 and Neurons without noticeable differences. On the other hand, FTH1 was significantly expressed in MSCs-FTH1 and Neurons-FTH1 cells, and the expression levels were not significantly different. The R2 value was significantly increased in MSCs-FTH1 and Neurons-FTH1 cells, which was consistent with the findings of Prussian blue staining, transmission electron microscopy, and intracellular iron measurements. These results suggest that FTH1 gene expression did not affect MSC differentiation into neurons and was not affected by neural differentiation. Thus, MRI reporter gene imaging based on FTH1 can be used for the detection of neurally differentiated cells from MSCs.

## 1. Introduction

Mesenchymal stem cells (MSCs) exhibit pluripotency and have been extensively applied in preclinical and clinical studies of many types of human diseases in recent years [1–4]. In particular, studies on the application of MSCs in neurological diseases are a hotspot [5–8]. The common neurological diseases are mainly caused by loss or damage of neurons or glial cells. The proliferation and neural differentiation potentials of stem cells can be harnessed to promote the regeneration of nervous tissues to achieve the purpose of organ or tissue repair [9, 10].

During the process of stem cell transplantation therapy, real-time dynamic monitoring of the distribution, migration, proliferation, and differentiation of transplanted cells should be performed. At present, imaging methods for cell tracing mainly include optical imaging [11], nuclear medicine imaging [12], and magnetic resonance imaging (MRI) [13, 14]. Given the advantages of increased spatial resolution, excellent soft tissue contrast, and lack of irradiation, MRI is highly valuable [15].

It is impossible to directly distinguish between transplanted cells and host cells using the existing MRI resolution. Therefore, some imaging mediators must be introduced into cells in advance to enhance the sensitivity of MRI in the display of cells. Previous studies mainly used superparamagnetic iron oxide (SPIO) nanoparticles to label cells [16–18]. Although this method has the advantages of high labeling efficiency and easy operation, it also has inherent deficiencies. The level of iron particles in cells gradually decreases as cells proliferate; therefore, the long-term tracing of transplanted cells cannot be achieved [19–21]. MRI reporter imaging can overcome this deficiency. The principle is to introduce a reporter gene into cells. Through the sustained expression and iron accumulation effect of the reporter gene, cells will produce significant MRI signal changes. Current MRI reporter genes mainly include transferrin receptor [22], tyrosinase [23], *β*-galactosidase [24], MagA gene [25, 26], and ferritin gene [27]. Among them, the ferritin gene as an endogenous reporter gene has relatively more applications in the studies of MRI reporter gene imaging. The ferritin gene includes two subunits: light chain and heavy chain (FTH1). FTH1 exhibits stronger ferrous oxidase activity and can promote the absorption of surrounding iron ions by cells.

Currently, FTH1 has been applied as an MRI reporter gene in tracing various cell types [28, 29]. The majority of the study results indicate that because the FTH1 gene is integrated into the cell genome, its expression is not affected by cell division and proliferation [29–31]. However, it is unclear whether FTH1 expression is affected by the neural differentiation of stem cells and whether it can affect cell differentiation. The aim of this study was to explore the relationship between FTH1 expression and neural differentiation in the differentiation of MSCs carrying FTH1 into neuron-like cells and to investigate the feasibility of using FTH1 as an MRI reporter gene to detect neurally differentiated cells.

## 2. Materials and Methods

### 2.1. Acquisition and Identification of MSCs

All animal protocols performed in this study were approved by the Animal Care and Use Committee of Chongqing Medical University, and the procedures were conducted in compliance with the National Institutes of Health Guidelines. All efforts were made to minimize animal suffering.

Four-week-old Sprague-Dawley (SD) rats (40-60 g) were used for isolation and culture of MSCs according to previous methods [21]. Briefly, the long bones of four limbs of rats were isolated. The bone marrow cavity was washed with Dulbecco's Modified Eagle's Medium (DMEM; Gibco, Grand Island, NY, USA), and cells were centrifuged and resuspended in the washing solution. Cells were cultured in DMEM/F12 supplemented with 10% fetal bovine serum (FBS; HyClone, Logan, UT, USA), 100 units/ml penicillin, and 100 g/ml streptomycin at 37°C under 5% CO2. Third generation cells with excellent growth status were collected, digested in 0.25 trypsin, centrifuged at 4°C (1000 r/min, 5 min), washed with phosphate-buffered saline (PBS) thrice, and stored for future use.

For identification of stem cells, the monoclonal antibodies CD29, CD90, CD34, and CD45 (Beyotime, Nanjing, Jiangsu, China) were separately added to the cells and incubated in the dark for 30 min. Cells were washed with PBS thrice, resuspended in 200 *μ*l of PBS, detected, and analyzed using a flow cytometer.

### 2.2. Construction of the Recombinant Lentivirus Carrying FTH1

The cDNA sequence of the FTH1 gene (accession number BC000857) was synthesized (primers: forward, AACCGTCAGATCGCACCGGTGCCACCATGACGACCGCGTCCACCTC; reverse, TCCTT GTAGTCCATGAATTCGCTTTCATTATCACTGTCTC). After polymerase chain reaction (PCR) amplification, the DNA fragment was double digested with EcoR1 and AgeI, purified, and ligated to the empty lentiviral vector CMV-MCS-3Flag-Puro containing the CMV promoter to obtain the recombinant lentiviral plasmid CMV-FTH1-3Flag-Puro (pLV-FTH1). After PCR and DNA sequencing, pLV-FTH1, the packaging plasmid pHelper 1.0, and the envelope plasmid pHelper 2.0 were cotransfected into 293T cells (Jikai Gene Technology, Shanghai, China) to obtain the recombinant lentivirus carrying the FTH1 gene, which was referred to as LV-FTH1. The virus titer was determined using enzyme-linked immunosorbent assay (ELISA).

### 2.3. Reporter Gene Transduction and Cell Identification

To determine the optimal multiplicity of infection (MOI), lentivirus at various MOIs (0, 5, 10, 20, 30, 50) was used to infect the MSCs. After 48 h of infection, the infection efficiency was detected by flow cytometry. The cell viability after 48 h of infection at different MOIs was also evaluated using Cell Counting Kit-8 (CCK-8, Jikai Gene Technology, Shanghai, China) assays. Briefly, 10 *μ*l of CCK-8 reagent was added to each group of cells and incubated for 2 h. The optical density (OD) values (OD_450_ and OD_650_) were measured using an optical density scanner. The final OD value was calculated as follows: OD=OD_450_–OD_650_.

When the cell confluence reached 30-40%, the lentivirus LV-FTH1 at the optimal MOI was added. After 24 h, the culture medium was replaced, and cells were continuously cultured. After 48 h, 4 *μ*g/ml of puromycin was added for selection for 7 d. The MSCs carrying FTH1 that contained the drug resistant gene were obtained and were named MSCs-FTH1.

A total of 1×10^5^ MSCs-FTH1 cells were collected. An equal number of cells transduced with the empty virus (MSCs-LV) and MSCs not transduced with virus were used as controls. FTH1 gene expression was detected using Western blot. The total cellular protein in the 3 groups was extracted using a cellular total protein reagent kit (Beyotime, Nanjing, Jiangsu, China). The protein concentration was measured using the bicinchoninic acid (BCA; Beyotime, Nanjing, Jiangsu, China) method. After SDS-polyacrylamide gel electrophoresis (SDS-PAGE; Beyotime, Nanjing, Jiangsu, China), proteins were transferred onto a polyvinylidene fluoride (PVDF) membrane (Jikai Gene Technology, Shanghai, China). The membrane was blocked in 5% bovine serum albumin (BSA, Sigma-Aldrich, St. Louis, MO, USA) for 1 h and incubated with the primary rabbit anti-FTH1 monoclonal antibody (1 : 1000, Abcam, Cambridge, MA, UK) or mouse anti-*β*-actin monoclonal antibody (1 : 1000, Abcam, Cambridge, MA, UK) at 4°C overnight. The membrane was then incubated with the secondary goat anti-rabbit (1 : 5,000) or goat anti-mouse (1 : 1000) antibody (Genscript, Nanjing, Jiangsu, China) for 2 h. Finally, the results were developed using the Enhanced Chemiluminescence (ECL) Reagent Kit (Beyotime, Nanjing, Jiangsu, China). To further confirm the expression of FTH1, the tag protein Flag in 3 types of cells was also detected via Western blotting with the primary mouse anti-Flag antibody (1 : 2000) and the secondary goat anti-mouse antibody (1 : 2,000) (Jikai Gene Technology, Shanghai, China).

In addition, the expression of the Flag protein was detected using immunofluorescence. Cells were fixed in 4% paraformaldehyde for 20 min, permeabilized in 1% Triton X-100 solution at room temperature for 10 min, and blocked in 5% BSA at room temperature for 1 h. Then, the cells were incubated with the primary antibody (mouse anti-Flag 1 : 200) and the secondary antibody (anti-mouse Cy-3 conjugated, 1 : 1000), counterstained with 4,6-diamidino-2-phenylindole (DAPI; Beyotime, Nanjing, Jiangsu, China), and observed under a fluorescence microscope (TE2000-S, Nikon, Japan).

### 2.4. *In Vitro *Differentiation of MSCs into Neurons and Identification

MSCs-FTH1 cells with excellent growth were inoculated into a 24-well plate. When the cell confluence reached 70-80%, 1 mmol/L all-trans retinoic acid (ATRA; Beyotime, Nanjing, Jiangsu, China) was added for preinduction for 24 h. The original culture medium was discarded. Cells were washed with PBS thrice, and modified neuronal medium (MNM, DMEM/F12/1.6% DMSO/160 M butylated hydroxyanisole/20 mM KCl/1.6 mM valproic acid/8 M forskolin/0.8 M hydrocortisone/4 g/ml insulin) was added for induction for 24 h [32]. The obtained cells were neuron-like cells and were named Neurons-FTH1. MSCs were induced into neuron-like cells using the above method and were named Neurons and used as the control.

The expression of neuron-specific markers nestin, neuron-specific enolase (NSE), and microtubule-associated protein (MAP-2) in Neurons-FTH1 and Neurons was detected using immunofluorescence. Cells were fixed in 4% paraformaldehyde for 20 min, permeabilized in 1% Triton X-100 at room temperature for 10 min, and blocked in 5% BSA at room temperature for 1 h. Cells were incubated with the primary mouse anti-NSE monoclonal antibody, rabbit anti-nestin monoclonal antibody, or rabbit anti-MAP2 monoclonal antibody (1 : 200, Proteintech, IL, USA) at 4°C overnight. Next, cells were incubated with the appropriate secondary goat anti-mouse or goat anti-rabbit DyLight 488- or DyLight 594-conjugated antibody (1 : 1000, Proteintech, IL, USA), counterstained with DAPI, and observed under a fluorescence microscope (TE2000-S, Nikon, Tokyo, Japan).

In the NSE immunofluorescence image, 10 fields were randomly selected to count the NSE positive and axon forming cells. Its ratio with the total number of cells was the differentiation rate. In addition, the length of axons of differentiated cells was measured using ImageJ software (National Institutes of Health, Bethesda, Maryland, USA). The measurement was independently performed thrice, and each measurement was repeated thrice.

The expression of nestin, NSE, and MAP-2 before and after cell differentiation was quantitatively measured using quantitative real-time PCR (qRT-PCR). Total cellular RNA was extracted using TRIzol (Invitrogen, Carlsbad, CA, USA). RNA was reversely transcribed into cDNA using the PrimeScript RT Reagent Kit (TaKaRa, Tokyo, Japan). Using each synthesized cDNA as a template, PCR was performed with the following primers: Nestin: 5′-AGCTGGCG CACCTCAAGATG-3′ and 5′-AGGGAAGTTGGGCTCAG GAC-3, NSE: 5′-TCGCCACATTGCTCAACT-3′ and 5′-AACTCAGAGGCAGCCACATC-3′, and MAP2: 5′-AATCAGCTCTGGCTCCCAGT-3′ and 5′-AGTGGGTGTTGAGGTACCAC-3′. A total of 30 cycles was performed.

### 2.5. Western Blot Detection of FTH1 Expression before and after Cell Differentiation

Western blotting was performed to detect FTH1 expression in MSCs and MSCs-FTH1 before induction and in Neurons and Neurons-FTH1 after 48 h of induction. The total cellular protein was extracted, and the protein concentration was determined using the BCA method. After SDS-PAGE, proteins were transferred onto a PVDH membrane. The membrane was blocked in 5% BSA for 1 h, incubated with the primary anti-*β*-actin antibody or the primary anti-FTH1 antibody at 4°C overnight, and the secondary antibody for 2 h. Finally, the results were developed using ECL. Each group of experiments was repeated thrice.

### 2.6. Detection of Cell Viability before and after Neural Differentiation

MSCs and MSCs-FTH1 before differentiation and Neurons and Neurons-FTH1 after differentiation were cultured for 72 h in the presence or absence of 500 *μ*M ferric ammonium citrate (FAC; Beyotime, Nanjing, Jiangsu, China). The effect of FTH1 expression and/or iron ions on cell viability was detected using a CCK-8 assay. Each group had 5 replicated wells. Briefly, 10 *μ*l of CCK-8 reagent was added to each well. The plates were gently mixed and incubated for 2 h. The OD values (OD_450_ and OD_650_) of each well were measured using an optical density scanner. The final optical density value of each well was calculated as follows: OD=OD_450_–OD_650_. Each well was plated in triplicate.

### 2.7. *In vitro* MR Imaging of Cells

The four groups of cells (MSCs, MSCs-FTH1, Neurons, and Neurons-FTH1) were cultured in the presence of 500 *μ*M FAC for 72 h. A total of 1×10^6^ cells were collected, washed with PBS thrice, resuspended, and transferred into a 0.5-ml PCR tube. The tube was left standing for 30 min to form the cell pellet at the bottom of the tube. Cells were scanned in an Achieva 3.0 T MRI scanner (Philips Medical Systems, Eindhoven, Netherlands) using the knee joint coil. The scanning sequences and parameters were as follows: T2WI: TR=2000 ms, TE=65 ms: GRE sequence: TR=2000 ms, TE: 13, 26, 39, 52, 65, and 78 ms, FOV=120×120 mm, matrix=512×512, and slice thickness=1 mm. The R_2_ artificial color map (R_2_ map) was obtained using the R_2_ star software in the postprocessing workstation. Five different regions of interest (ROIs, 3.0 mm^2^) were selected for measuring R_2_ values.

### 2.8. Detection of Intracellular Iron

*Prussian Blue Staining*. Cells were fixed in 4% paraformaldehyde for 20 min and stained with the Prussian blue staining reagent (mixture of 2% potassium ferrocyanide and 2% HCl at an equal volume) at room temperature for 30 min and nuclear fast red staining solution for 2-3 min. After washing with ddH2O, cells were mounted in neutral balsam and observed under a light microscope.

*Transmission Electron Microscopy (TEM)*. Cells were fixed in 2.5% glutaraldehyde solution and 1% osmic acid for 30 min and stained in 0.5% uranyl acetate overnight. Cells were dehydrated in a series of ethanol gradations, immersed and embedded into the mixture of the equal volume of 100% acetone and the EP812 embedding solution, ultra-thin sectioned at 100 nm, and observed under a Hitachi-7500 transmission electron microscope (Hitachi, Tokyo, Japan).

*Detection of Intracellular Iron Content*. A total of 1×10^5^ cells from each group were centrifuged, and the pellet was dried at 110°C overnight. Cells were digested in 500 *µ*l of a perchloric acid-nitric acid mixture (1 : 3) at 60°C for more than 3 h to completely dissolve the cells. The iron ion concentration was measured using an atomic absorption spectrophotometer. The results were expressed as pg/cell. Each sample was measured 3 times.

### 2.9. Statistical Methods

All data were expressed as $\bar{x}$±SD. The comparison of the neural differentiation rates between the MSCs and MSCs-TFH1 groups was performed using the chi square test. The axonal lengths between the Neurons and the Neurons-FTH1 groups were compared using Student's t test. The comparison of mRNA levels of neuron-specific markers, FTH1 gene expression, cell viability, R2 values, and iron levels among the MSCs, MSCs-FTH1, Neurons, and Neurons-FTH1 groups was performed using analysis of variance (ANOVA). The pair-wise comparison was performed using the LSD method. The Statistical Package for the Social Sciences software version 19.0 (SPSS Inc., Chicago, IL, USA) was used for statistical analyses. P<0.05 indicated statistical significance.

## 3. Results

### 3.1. Identification of MSCs

**Figure 1:**
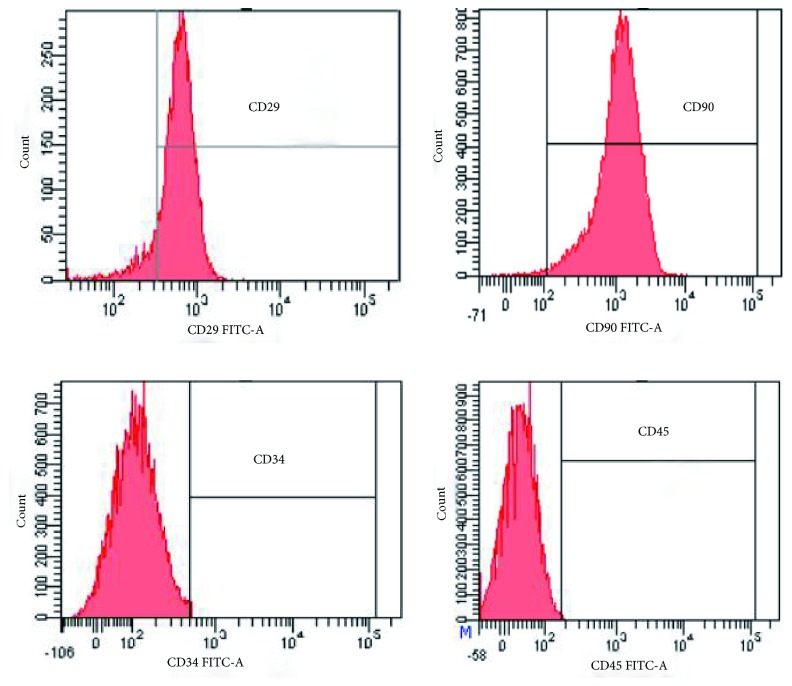
Detection of surface markers of MSCs using flow cytometry. The MSCs specific markers, CD29 and CD90, had strong positive expression, while CD34 and CD45 did not have obvious positive expression.

The positive rates of the surface markers of the 3^rd^ generation MSCs detected by flow cytometry were 96.7% and 99.2% for CD29 and CD90, respectively; however, the CD34 and CD45 positive rates were only 0.2% and 0.1%, respectively (Figure 1).

### 3.2. Transduction of MSCs with the FTH1 Gene and Identification

**Figure 2:**
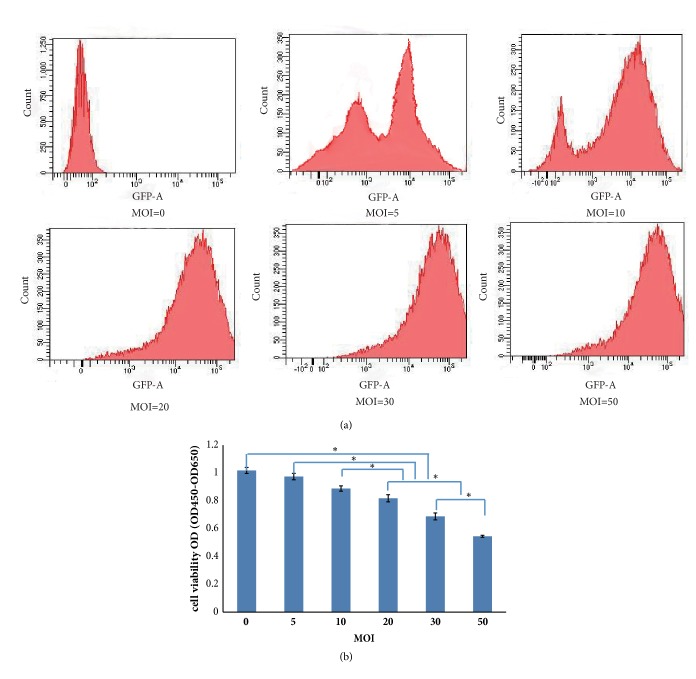
Infection efficiency and cell viability. (a) Flow cytometry of the infection efficiency of lentivirus at different MOIs. When the MOI increased, the infection efficiency increased. When the MOI was 10, the infection efficiency reached 78.5%. (b) CCK-8 detection of the cell viability at different MOIs. The cell viability after transduction with lentivirus was significantly decreased in the groups treated at a MOI≥20 (P<0.05) but was not decreased significantly in the groups with a MOI≤10 compared with the control (P>0.05) (*∗* indicates a significant difference among groups treated at different MOIs).

The optimal MOI was determined by the infection efficiency and cell viability. When the MOI increased, the infection efficiency increased. When the MOI reached 10, the infection efficiency reached 78.5% (Figure 2(a)). CCK-8 assay results showed that the cell viability did not decrease significantly in the groups treated at a MOI≤10 but decreased significantly in the groups with a MOI≥20 compared with the control (P<0.05) (Figure 2(b)). Considering cell safety, the optimal MOI was determined to be 10, although the infection efficiency continued to increase when the MOI exceeded 10. Using this protocol, MSCs carrying the reporter gene FTH1 were successfully obtained.

**Figure 3:**
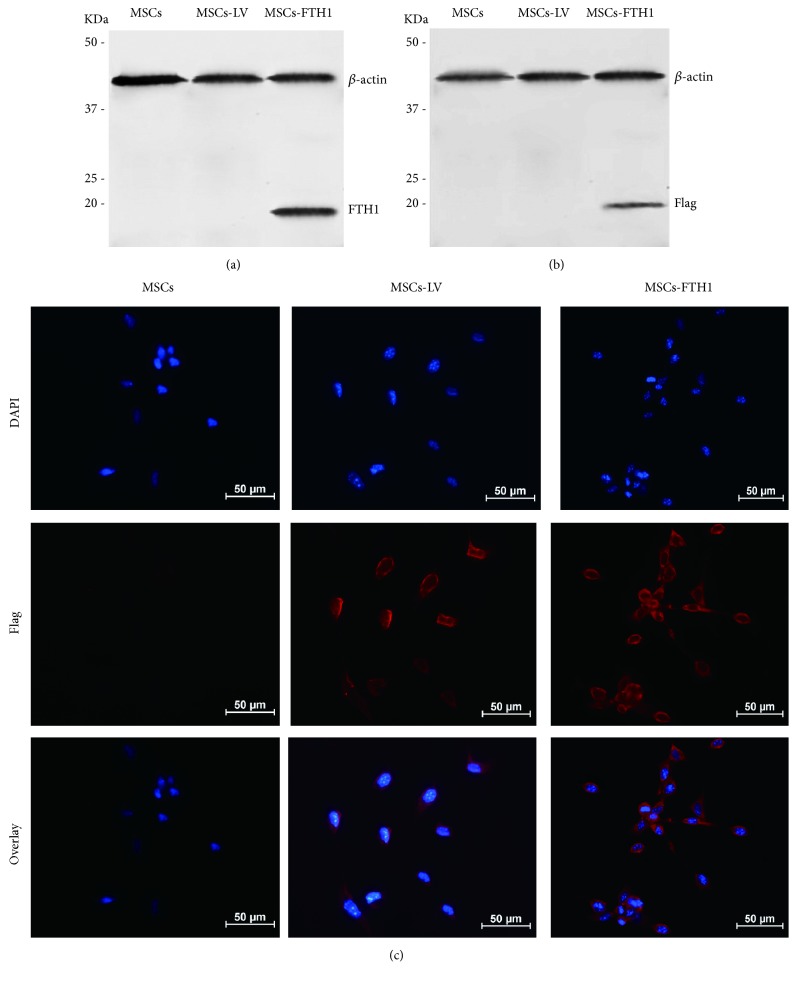
FTH1 and Flag-tag expression in MSCs. (a) Detection of the FTH1 gene in MSCs via Western blot. MSCs-FTH1 exhibited a positive protein band at 21 KDa, which was consistent with the theoretical size of the FTH1 protein. The positive band was not observed in MSCs and MSCs-LV in the control group. (b) Detection of the Flag-tag in MSCs via Western blot. A positive band near FTH1 was observed, which was of the expected molecular weight of the recombinant FTH1 (21 KDa) and Flag (1 KDa) proteins. (c) Detection of the Flag protein in MSCs using immunofluorescence. Red fluorescence was observed in MSCs-FTH1 and MSCs-LV but not in MSCs. These results confirmed that transduction with TFH1 was successful.

Western blotting results revealed that MSCs transduced with lentiviruses carrying the FTH1 gene (MSCs-TFH1) exhibited a positive band at 21 KDa, which was consistent with the theoretical size of the FTH1 protein. The positive band was not observed in the MSCs and MSCs-LV in the control groups (Figure 3(a)). Western blotting of the tag protein Flag also showed a positive band near FTH1 (Figure 3(b)), which was of the expected molecular weight of the recombinant FTH1 (21 KDa) and Flag (1 KDa) proteins. Immunofluorescence revealed that the Flag protein was expressed in MSCs-TFHI and MSCs-LV but was not expressed in MSCs (Figure 3(c)). The above results confirmed that MSCs were was successfully transduced with FTH1.

### 3.3. Morphological Observation and Quantitative Analyses of MSCs before and after Neural Differentiation

**Figure 4:**
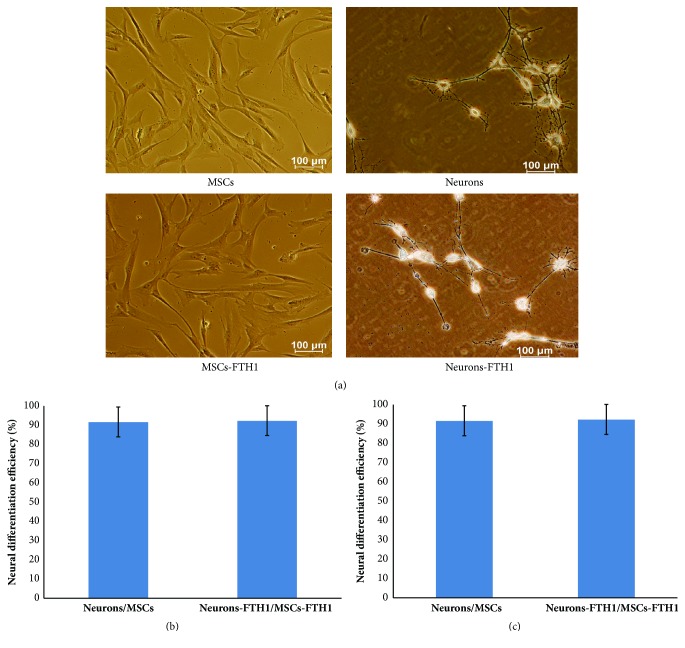
Differentiation of MSCs into neuron-like cells. (a) Before induction, MSCs and MSCs-TFH1 exhibited a flat or spindle shape and no refraction. After induction, cells had enhanced transparency and thin and long processes that exhibited bipolar or multiple-polar expansion and connected to adjacent cells. (b) The neural differentiation rates between MSCs and MSCs-FTH1 did not exhibit significant differences (P>0.05). (c) The mean axonal lengths between Neurons and Neurons-FTH1 did not exhibit significant differences (P>0.05).

Before differentiation induction, MSCs and MSCs-FTH1 exhibited a flat or spindle shape and did not have refraction. After ATRA preinduction and MNM induction for 24 h, cell morphology exhibited significant changes. Most cells had enhanced transparency. In addition, the cytoplasm shrank to the nuclear center, and cells had long and thin processes that exhibited bipolar or multipolar expansion to the surroundings, formed secondary or even multiple levels of processes, and connected to adjacent cells (Figure 4(a)). The neural differentiation rates of MSCs and MSCs-TFH1 were (91.56±7.89)% and (92.23±7.64)%, respectively, and these cell types did not exhibit significant differences (P>0.05) (Figure 4(b)). The mean axonal length of Neurons was (97.13±10.89), which was not significantly different from Neurons-FTH1 (102.24±12.61) (P>0.05) (Figure 4(c)).

### 3.4. Expression of Neuron-Specific Markers before and after Neural Differentiation

Immunofluorescence results showed that undifferentiated MSCs and MSCs-FTH1 did not express neuron-specific markers. After cells were induced to differentiate into Neurons and Neurons-FTH1, strong positive expression of NSE, Nestin, and MAP-2 was detected (Figure 4(a)).

**Figure 5:**
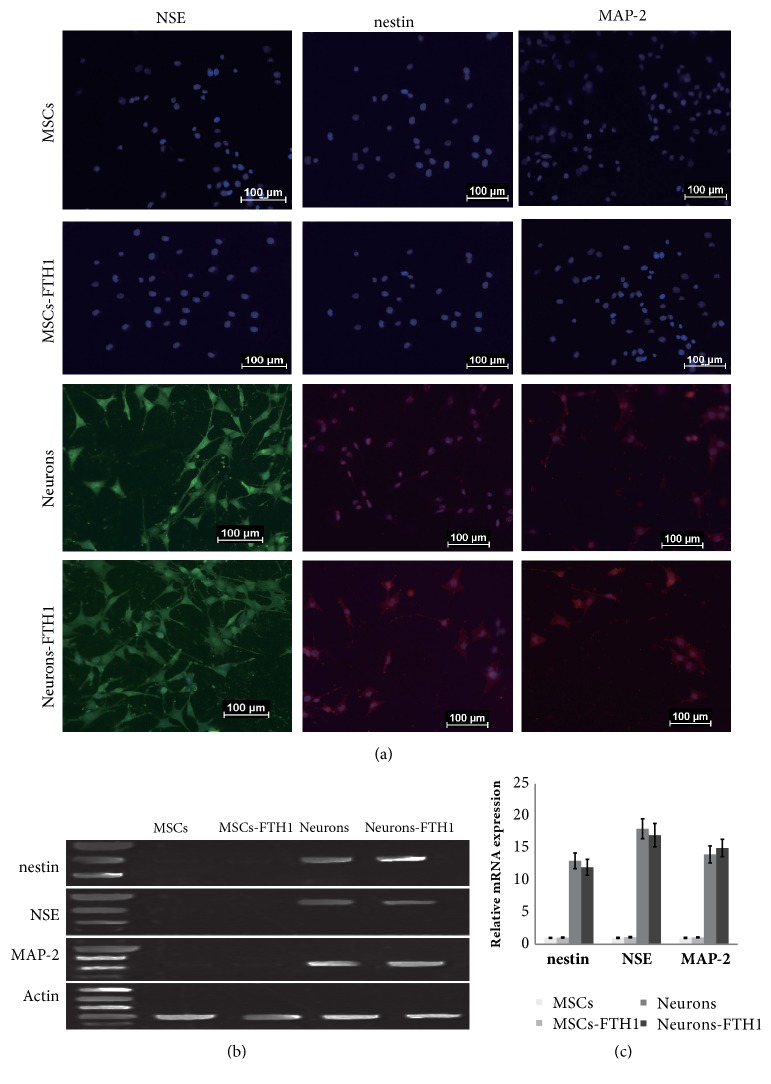
Expression of neuron-specific markers before and after differentiation. (a) Immunofluorescence results for nestin, NSE, and MAP-2 in MSCs and MSCs-TFH1 before induction were negative. After induction, 3 markers in Neurons and Neurons-FTH1 exhibited strong positive expression. (b)-(c) qRT-PCR demonstrated that nestin, NSE, and MAP-2 were not significantly expressed in MSCs and MSCs-FTH1 before induction. After differentiation induction, nestin, NSE, and MAP-2 were significantly expressed in Neurons and Neurons-FTH1, and no significant differences were noted between these two groups (P>0.05).

qRT-PCR quantitative detection of neuron-specific markers before and after cell differentiation revealed that nestin, NSE, and MAP-2 were not expressed in MSCs and MSCs-FTH1 before differentiation. After differentiation induction, nestin, NSE, and MAP-2 were obviously expressed in Neurons and Neurons-FTH, and the difference between these two groups was not significantly different (P>0.05) (Figures 5(b) and 5(c)).

### 3.5. FTH1 Expression in MSCs before and after Differentiation

**Figure 6:**
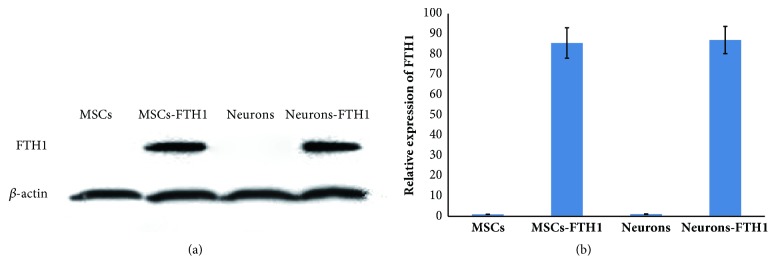
FTH1 expression in cells before and after differentiation. (a) Western blot results revealed that MSCs and Neurons did not exhibit a target band, whereas MSCs-FTH1 and Neurons-FTH1 both exhibited a significant target band. (b) The gray density analysis results showed that the gray density ratio between MSCs-FTH1 and Neurons-FTH1 did not exhibit significant differences (P>0.05).

Western blot results revealed that MSCs and Neurons that did not contain the FTH1 gene did not exhibit a target band, whereas both MSCs-FTH1 and Neuron-FTH1 exhibited an obvious target band (Figure 6(a)). The gray density analysis results revealed that the gray density ratio between MSCs-FTH1 and Neurons-FTH1 did not exhibit a significant difference (P>0.05) (Figure 6(b)). These results indicate that FTH1 gene expression was not significantly altered after MSCs were induced to differentiate into neuron-like cells.

### 3.6. The Effect of FTH1 Expression on Cell Viability

**Figure 7:**
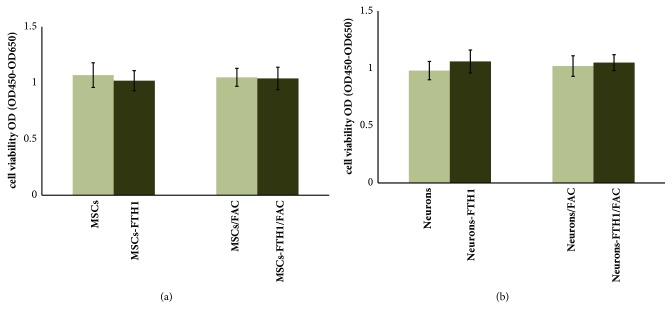
Detection of cell viability using CCK-8. (a) The OD values (OD_450_-OD_650_) among 4 groups of cells (MSCs, MSCs-FTH1, MSCs/FAC, and MSCs-FTH1/FAC) before differentiation did not have significant difference, P>0.05. (b) The OD values among 4 groups of cells (Neurons, Neurons-FTH1, Neurons/FAC, and Neurons-FTH1/FAC) after differentiation did not exhibit significant differences, P>0.05.

CCK-8 detection results showed that the OD values (OD_450_-OD_650_) of the 4 groups of cells (MSCs, MSCs-FTH1, MSCs/FAC, and MSCs-FTH1/FAC) before differentiation were 1.07±0.11, 1.02±0.09, 1.05±0.08, and 1.04±0.10, respectively; P>0.05 (Figure 7(a)). The OD values of the 4 groups of cells (Neurons, Neurons-FTH1, Neurons/FAC, and Neurons-FTH1/FAC) after differentiation were 0.98±0.08, 1.06±0.10, 1.02±0.09, and 1.05±0.07, respectively; P>0.05 (Figure 7(b)). These results suggested that FTH1 gene expression and 500 *μ*M FAC both did not have significant effects on cell viability before and after differentiation.

### 3.7. *In vitro *MRI Findings

**Figure 8:**
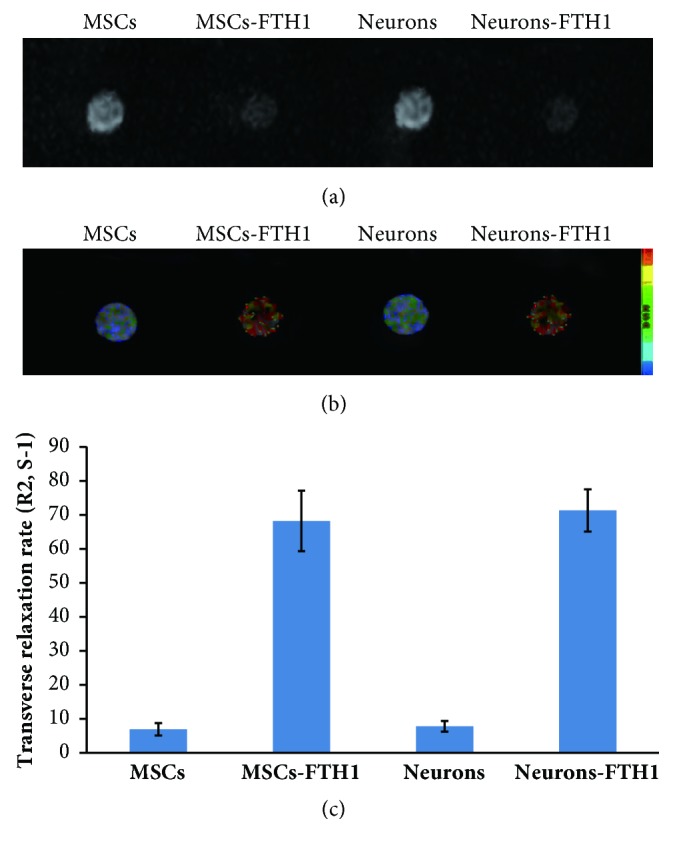
MR imaging of cells* in vitro*. (a) T_2_WI revealed that the signals in MSCs-FTH1 and Neurons-FTH1 significantly decreased compared with MSCs and Neurons. (b)-(c) R2 map and R2 value measurement revealed that R2 values in MSCs-FTH1 and Neurons-FTH1 were significantly increased compared with MSCs and Neurons-FTH1 (P<0.05). However, no significant differences were noted between MSCs-FTH1 and Neurons-FTH1 (P>0.05).

MRI scanning was performed on MSCs-FTH1, Neurons-FTH1, MSCs, and Neurons after 72 h of culture in the presence of 500 *μ*M FAC. The results showed that the precipitation of T_2_WI signals in MSCs-FTH1 and Neurons-FTH1 significantly decreased compared with MSCs and Neurons (Figure 8(a)). The R2 map quantitative measurement showed that the R2 values of MSCs-FTH1 and Neurons-FTH1 were significantly increased compared with MSCs and Neurons-FTH1, and the difference between MSCs-FTH1 and Neurons-FTH1 was not significant (Figures 8(b) and 8(c)). These results confirmed that the iron transport function of FTH1 was clear and was not affected by neural differentiation.

### 3.8. The Iron Accumulation Effect of FTH1

**Figure 9:**
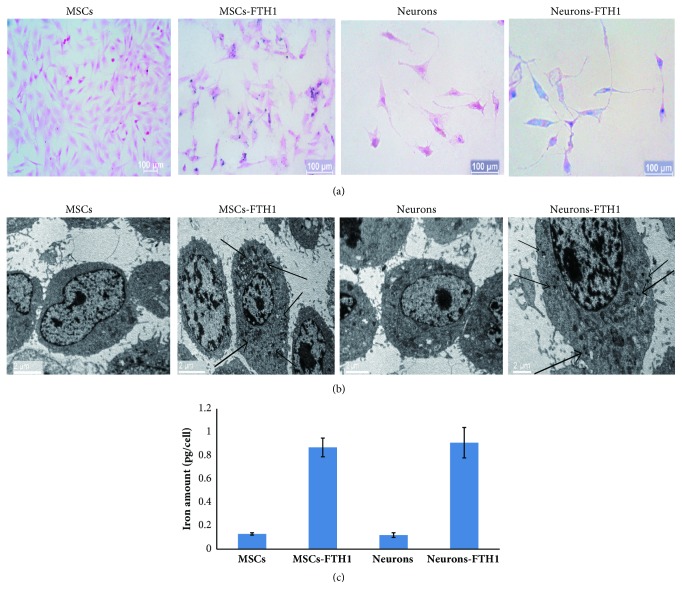
The iron transport effect of FTH1. (a) Prussian blue staining: there was an increased number of blue particles in the cytoplasm of both MSCs-FTH1 and Neurons-FTH1, whereas no obvious blue particles were observed in MSCs and Neurons in the control group. (b) Transmission electron microscopy: the cytoplasm of MSCs-FTH1 and Neurons-FTH1 contained black electron-dense particles (arrows), whereas the cytoplasm of MSCs and Neurons in the control group did not contain electron-dense particles. (c) Atomic absorption spectrophotometer detection revealed that the iron levels between MSCs-FTH1 and Neurons-FTH1 were not significantly different (P>0.05), but the levels were increased compared with MSCs and Neurons (P<0.05).

The Prussian blue staining results showed that after 4 groups of cells were cultured in the presence of FAC for 3 d, the cytoplasm of MSCs-FTH1 and Neurons-FTH1 exhibited more blue particles, whereas MSCs and Neurons in the control group did not exhibit obvious blue particles (Figure 9(a)). The transmission electron microscopy results were consistent with the Prussian blue staining results. The cytoplasm of MSCs-FTH1 and Neurons-FTH1 contained black electron-dense particles, whereas the cytoplasm of MSCs and Neurons in the control group did not contain electron-dense particles (Figure 9(b)). The atomic absorption spectrophotometer detection results revealed that the iron levels in MSCs-FTH1 and Neurons-FTH1 were 0.87±0.08 pg/cell and 0.91±0.13 pg/cell, respectively, and there was no significant difference (P>0.05). However, the levels were significantly increased compared with those in MSCs (0.13±0.01 pg/cell) and Neurons (0.91±0.13 pg/cell) (P<0.05) (Figure 9(c)).

## 4. Discussion

The application of the MRI reporter gene imaging strategy depends on whether the reporter gene can be persistently and stably expressed in cells [30, 31]. This study successfully induced the differentiation of MSCs carrying the FTH1 reporter gene into neuron-like cells. The results demonstrated that the reporter gene was clearly expressed before and after differentiation and induced the iron accumulation effect, which could be displayed by a clinical 3.0T MRI scanner. These results provide important foundations for further use of the MRI reporter gene imaging for long-term tracing of the biological behaviors of MSCs after transplantation.

Safety is a prerequisite for the application of the MRI tracing strategy for stem cells [33, 34]. Previous studies demonstrated that although the use of SPIO labeling did not affect the proliferation activity of stem cells, it might reduce the osteogenic differentiation ability [35]. For the FTH1 reporter gene used as an MRI tracer, whether its expression affects stem cell differentiation and the effect of stem cell differentiation on the expression of the reporter gene should be confirmed. This study used a lentiviral vector to transduce the reporter gene FTH1 into MSCs. Under certain induction conditions, MSCs were successfully differentiated into neuron-like cells. Regardless of the morphological observation or quantitation of the differentiation efficiency and axonal lengths, MSCs with or without FTH1 transduction did not exhibit significant differences. The quantitative detection of several neuron-specific markers also did not reveal differences between these two groups. These results indicated that transduction with FTH1 did not have significant effects on the neural differentiation potential of MSCs. On the other hand, although differentiated neuron-like cells exhibit changes in morphology and various specific markers, FTH1 expression in cells was normal, and the expression levels did not exhibit significant differences compared with those before differentiation. These results suggest that the neural differentiation of stem cells also did not have significant effects on the expression of the reporter gene.

The greatest advantage of FTH1 reporter gene imaging involves the use of a lentiviral vector to transduce cells with the gene and to integrate the gene into the cell genome. Through the expression of the reporter gene, cells continuously accumulate iron to achieve the purpose of the long-term continuous tracing of cells. This overcomes the deficiency of the direct SPIO labeling method given that the iron level gradually decreases in cells as a result of division and proliferation. Previous studies demonstrated that MRI detection of SPIO-labeled stem cells* in vitro* and* in vivo* is limited to within 4 w and 2 w, respectively [19, 21]. Because iron in cells is not stable, and cells can be engulfed by macrophages, iron-induced MRI signal changes cannot truly reflect the number of cells [36, 37]. Although the iron transporting efficiency of FTH1 is relatively low, and the MRI signal changes are weak, the signals are stable and sustained. Therefore, theoretically, the strength of MRI signals can indirectly determine the number of transplanted cells [38]. The results of this study revealed that significant signal changes in 10^6^ FTH1-transduced cells could be discovered by 3T MRI, and the iron levels and MRI signal changes in cells before and after differentiation did not exhibit significant changes. These results indicated that the iron transporting effect of FTH1 was clear and stable. The sensitivity of MRI in cell imaging is not only associated with cell labeling methods and cell number but is also associated with the magnetic field intensity and imaging sequences [29, 30]. When the magnetic field intensity is increased, the MRI sensitivity is increased. The clinical 3T MRI used in this study could detect signal changes in 1×10^6^ cells. We speculate that MRI with increased field strength can detect fewer cells with more sensitivity. It is generally considered that the T2^*∗*^ sequence is more sensitive in the detection of the ferromagnetic material than T2. However, the former has the amplification effect and is easy to produce artifacts. In addition, its signal range and strength typically cannot truly reflect the number and distribution of cells [39]. Thus, we adopted the T2WI sequence in this study.

Currently, the effect of FTH1 gene expression and iron accumulation on cell viability remains controversial [40–43]. Some studies revealed that excessive expression of the FTH1 gene in cells results in the uptake of excessive unstable iron, which might induce the Fenton effect to cause inorganic oxidation of intracellular carboxylic acids, alcohols, and esters to damage cells [40, 41]. Other studies considered that the FTH1 gene catalyzes the reaction between stable divalent iron ions and intracellular hydroxyl radicals to produce ferric ions to attenuate the cytotoxic function of the Fenton effect induced by excessive uptake of exogenous irons [42, 43]. The inconsistency of these results might be associated with the experimental cell types and the concentration and time of supplemented iron. This study detected the cell viability before and after differentiation to determine the effect of two factors, namely, FTH1 gene expression and/or addition of iron ions, on cell viability. The results demonstrated that cell viability between MSCs and MSCs-FTH1 before neural differentiation and between Neurons and Neurons-FTH1 after differentiation both did not exhibit significant differences. In addition, regardless of before or after differentiation, the cell viability between these two groups of cells with or without the addition of 500 *μ*M FAC also did not exhibit significant differences, indicating that these two factors, namely, FTH1 gene expression and/or the addition of iron ions, did not have significant effects on cell viability.

This study confirmed the feasibility of using FTH1 in the imaging of neural differentiation of stem cells. However, the study has the following limitations. First, this experiment is limited to* in vitro* studies. To validate the iron accumulation function of FTH1, a certain concentration of FAC was added to the culture medium. The actual iron accumulation function of FTH1 in the body mainly depends on the iron uptake from surrounding tissues. Compared with the simple and controllable* in vitro* environment, the* in vivo* environment is more complex. Under this condition, some issues still await further* in vivo* experiments for confirmation, including whether the endogenous iron level can meet the requirement of MRI imaging, whether gene expression and iron accumulation in cells affect cell differentiation, and whether the reporter gene in cells can be continuously expressed after differentiation. Next, during the actual application in transplanted cell tracing, both stem cells and differentiated cells exhibited reporter gene expression and reduced MRI signals. Hence, simply relying on the MRI reporter gene imaging cannot distinguish cells before and after differentiation. Therefore, how to use MRI to determine whether stem cells have already been successfully differentiated into specific cells* in vivo*, i.e., using MRI to monitor the occurrence of the differentiation event of stem cells, has become a difficult problem in this research field and is a future direction of our work.

## 5. Conclusions

This study successfully differentiated MSCs carrying the FTH1 gene into neuron-like cells. FTH1 gene expression neither affected the differentiation of MSCs into neurons nor was affected by neural differentiation. The iron accumulation effect was sufficient to induce changes in 3T MRI signals. These results suggested that MRI reporter gene imaging based on FTH1 could be used for the detection of neurally differentiated cells from MSCs.

## Data Availability

The data are available from the corresponding author upon request.
